# The Interplay of Relational and Non-relational Processes in Sentence Production: The Case of Relative Clause Planning in Japanese and Spanish

**DOI:** 10.3389/fpsyg.2018.01573

**Published:** 2018-09-11

**Authors:** Laura Rodrigo, José M. Igoa, Hiromu Sakai

**Affiliations:** ^1^Department of Basic Psychology, Faculty of Psychology, Autonomous University of Madrid, Madrid, Spain; ^2^Research Unit of Brain Science of Language, Inference and Thoughts, Faculty of Science and Engineering, Waseda University, Tokyo, Japan

**Keywords:** sentence production, relative clauses, eye-tracking, Japanese, Spanish

## Abstract

Speech planning involves different steps in order to transform a conceptual message into speech. These include establishing structural relations among constituents (i.e., *relational information*), and selecting the appropriate lexical items to convey the intended message (*non-relational elements*). However, the precise way relational and non-relational information are computed when undertaking linguistic encoding is not clear. This paper explores how the pre-linguistic message undergoes linguistic encoding, and what kind of information (relational or non-relational) is prioritized in doing so. We analyze the production planning of Relative Clauses in Spanish (a head-initial language) and Japanese (a head-final language) by monolingual speakers, by means of the eye-tracking method while participants described colored pictures. Although in both Spanish and Japanese the structure under study is the same (with the same syntactic configuration), word order is entirely opposite between both languages. In Japanese, the head noun is not uttered until the end of the clause, thus making it possible to explore sentence planning in a structure where the syntactically most dominant element (the head noun, HN) is not the first element. Variables tested were type of relative clause, with either the agent or the patient as head noun, and the animacy of the agent and the patient of the event, the latter allowing the manipulation of the conceptual saliency of the elements involved. Results showed Japanese speakers focus extensively on the HN before directing their gazes to the element they are going to utter first, suggesting a speech planning process that prioritizes relational information, that is, structural scaffolding. Spanish monolinguals, in turn, showed a pattern in which both structural and linear information appear to be more closely related from the beginning. In both languages, the animacy of isolated elements had little effect on gaze patterns. Results point to a planning process that prioritizes structural relations over access to lexical elements in order in the planning of complex structures, with room for flexibility when the grammar of the language allows so.

## Introduction

In order to articulate a message, a speaker must undergo a series of stages: following the conceptual representation of the message, the speaker must choose a structural form for it, deciding on the grammatical relations among constituents (structural encoding) and choosing the lexical elements (lexical encoding) so as to arrange them in the precise word order in which the message will be uttered, before encoding the phonological form of the words and articulating them (Bock and Levelt, 1994; Ferreira, 2010). These stages take place incrementally, that is, speakers do not wait until the whole process in one stage has finished in order to proceed to the next stage. Rather, sentence planning unfolds in a piecemeal fashion: the planning process starts as soon as a piece of information becomes available. While a unit of information is being processed at a given stage, the next upcoming item begins to undergo the planning process at the previous stage. Thus, the first available information will be processed earlier, and as a consequence, it will regularly be uttered at an earlier and/or more prominent position in the sentence (Branigan and Feleki, 1999; Prat-Sala and Branigan, 2000).

The effects of conceptually or visually salient information–information that is more available when the message is being encoded during the planning process–on structural and lexical encoding in sentence production have raised the question of which of these processes precedes the other, and to what extent they overlap during sentence planning. Studies that have underscored the effects of the conceptual saliency of specific elements of the message on speech planning processes (animacy: McDonald et al., 1993; Branigan et al., 2008; imageability: Bock and Warren, 1985; contextual prominence: Prat-Sala and Branigan, 2000) point out that more salient elements are accessed earlier and, as a result, are placed earlier or are assigned a more prominent grammatical function (subject or topic) in the sentence. Thus, sentence planning may be viewed as an interplay of *relational* (i.e., structural, hierarchical) and *non-relational* (i.e., lexical, linear) processes (Konopka and Meyer, 2014). Relational processes are those concerning the establishment of grammatical relations between elements: processes involving the creation of the structural scaffold of the utterance (Bock and Ferreira, 2014)^1^. On the other hand, non-relational processes are those involved in selecting the proper lexical items that will be used in the sentence. The relative importance of relational and non-relational processes, and the precise moment at which each one becomes engaged in the planning process is a matter of debate, and seems to shift from one study to another.

Our aim in this article is to explore these issues by analyzing the timing of relational and non-relational processes in the production of complex structures. Accordingly, we manipulated the conceptual saliency (in this case, animacy) of the elements involved in the utterance, in order to analyze to what extent linguistic encoding processes are affected by the saliency of those elements from the point where the message is encoded. As we explain below, saliency was manipulated in the current experiments by means of the animacy of participants in the visual scenes that were used as prompts for sentence production. We explored these issues by making use of the eye-tracking methodology, which allows the online recording of eye movements and fixations supposedly concurrent with the underlying planning processes. Online measures like these purportedly enable us to tease apart the different processes involved in sentence planning as they unfold, distinguishing the temporal moment at which conceptual information (in this case, animacy), lexical access and structural planning are taking place.

Regarding these issues, there are two contrasting perspectives that define the positions currently represented in the literature: Hierarchical Incrementality accounts and Linear Incrementality accounts.

According to Hierarchical Incrementality accounts (e.g., Griffin and Bock, 2000; Bock et al., 2004; Ganushchak et al., 2014; Van de Velde et al., 2014), relational processes play an earlier and more definite role in utterance planning. In a pioneer work Griffin and Bock (2000) presented English speakers with pictures depicting transitive actions and had them describe them while monitoring their eye-movements. Their results showed that speakers first fixated equally both agent and patient of the scene, for a period of less than 400 ms. Only after this early period, speakers focused their gazes on the subject (the element they were going to utter first) and then, after speech onset, on the patient. The authors conclude that during this short period of 400 ms., which they dubbed “apprehension stage,” speakers are creating an early gist of the scene that will guide subsequent planning processes. This early stage includes the conceptual characteristics of the event, as well as the structural relations between the participants involved. The creation of this structure will subsequently guide the retrieval of lexical elements in a top-down fashion. The strong version of this position postulates that certain characteristics of the lexical items, such as their visual saliency, will not affect the planning process of the utterance (Griffin, 2004).

On the other hand, Linear Incrementality accounts (Gleitman et al., 2007; Brown-Schmidt and Konopka, 2008) rely more heavily on non-relational processes to explain the triggering of utterance planning. Gleitman et al. (2007) presented participants with different types of complex scenes, and asked them to describe them. Critically, they controlled saliency by means of marking either participant involved in the scene with a visual cue of which participants were unaware, prior to picture onset. They found that when the cue was effective in focusing gazes on the signaled element, participants were more likely to start their utterance with the cued element. Thus, they provided evidence that visual salience could guide planning, on a word by word basis, even in syntactically complex utterances: in this case, participants seemed to start articulation after the preparation of just one word, without a global apprehension of the scene. This view posits that planning will start with the first-to-be-uttered element, and only from there speakers will start building a structure, word by word. Critically, and contrary to hierarchical incrementality, linear incrementality assumes that the most salient element will be uttered first and, consequently, will be assigned its grammatical function first. Whilst hierarchical accounts assume that grammatical function is assigned before linear order, the opposite is true for linear incrementality accounts: the selection of the first element will result in the assignment of a grammatical function to that element. Similarly, the grammatical function of the following elements will be assigned only when the corresponding lexical items are retrieved in the same order as they are going to be uttered, thereby triggering the relational processes among selected elements.

Most studies have focused on sentences in which the first element and the subject coincide, making it difficult to disentangle the effects of relational and non-relational processes. However, recent studies controlling for the accessibility of relational and non-relational properties (e.g., Ganushchak et al., 2014; Van de Velde et al., 2014) point to the possibility that both structural and lexical features of messages are used from early stages of production planning. This kind of planning allows for some flexibility in prioritizing any of these features depending on task and contextual demands, along with the characteristics of the event and the elements to be expressed, so as to support a more efficient production process (Kuchinsky et al., 2011; Konopka and Meyer, 2014; Norcliffe and Konopka, 2015). In this sense, incrementality moves along a continuum between the two extremes of structurally- and lexically-guided planning (Norcliffe and Konopka, 2015).

In order to test these two positions against each other, we carried out a cross-linguistic comparison between two typologically different languages, Spanish and Japanese, in the production of grammatically complex sentences. Specifically, we used relative clauses, whose opposite word order in Spanish and Japanese would allow us to differentiate between Hierarchical and Linear Incrementality positions, as we will explain in due course. In addition, cross-linguistic studies have proved to be helpful in order to understand the mechanisms by which the interaction between processes occurs. Thanks to these studies we are beginning to understand that speech planning flexibility is not only dependent on the contextual demands of language production, but also on the particular characteristics of the grammar being used. Thus, differences in word order lead to differences in the time course of speech planning. For example, studies with languages in which the verb is placed at initial position–VOS languages like Tzeltal (Norcliffe et al., 2015), or Kaqchikel (Kubo et al., 2015) or VSO languages like Tagalog (Sauppe et al., 2013)– show that in these cases, speakers undertake the planning of structural relations among elements before deciding which element will be assigned the agent or the patient role in the sentence to be uttered. These languages additionally allow SVO sentences. In such cases, the time course of sentence formulation mimics that found in studies with canonical SVO languages like English (Griffin and Bock, 2000; Gleitman et al., 2007) or Dutch (Konopka and Meyer, 2014; Van de Velde et al., 2014). Not only word order has been shown to exert an influence on sentence formulation, but also other language-specific grammatical features may induce speakers to rely more on structural or on linear incremental strategies. In this regard, speakers of languages that possess grammatical function markers on lexical items are less likely to begin their utterance with the element that is first activated, and rely instead on a planning process guided by structural relations. In these languages, the grammatical function of the first lexical item must have been decided upon in order to start speech, thus making it difficult to initiate the assembly of grammatical relations right after the retrieval of the first lexical item (see Hwang and Kaiser, 2014, for Korean; Myachykov and Tomlin, 2008 for Russian; or Myachykov et al., 2011, for Russian and Finnish; additionally, Norcliffe et al. (2015) show similar effects in a verb initial language, Tzeltal). These results demonstrate the flexibility of ongoing processes in sentence formulation, as well as the various planning strategies available between and within languages.

The evidence presented above renders a picture of language production as a flexible process in which both linear and hierarchical incrementality play an important role. However, in the studies cited above, the subject, that is, the syntactically most dominant element, always precedes the object or patient, making it difficult to ascertain what kind of information is prioritized when undertaking linguistic encoding: either the access to single lexical items or the construction of a tentative structure. Likewise, in VOS languages, the initial position of the verb makes it difficult to interpret the initial gazes to either agent or patient^2^. With this question in mind, in the current study we aim to investigate from a cross-linguistic perspective what kind of information is prioritized when undertaking linguistic encoding, and to what extent conceptual saliency plays a role in this process. Importantly, we aim to explore what happens after the apprehension stage previously reported in the literature (that is, after the first 400 ms.), provided such stage is confirmed by our data. The apprehension period allegedly involves the activation of a conceptual representation of the event to be communicated, and hence precedes linguistic encoding. In the literature published so far, the first mentioned constituent always corresponds to the most syntactically dominant element, thus making it difficult to understand whether speakers are retrieving the lexical items to be placed in order or, conversely, are focusing on the syntactically most dominant element to create a syntactic scaffold of the sentence. In this study we aim to decide between both possibilities.

In order to address this question, and as was introduced before, we compared the production of relative clauses (hereafter RCs) in two typologically distant languages, Spanish and Japanese, by means of the eye-tracking methodology in a visual world paradigm. By monitoring participants' eye movements while they prepare and produce sentences, we expect to have a measure of which information is under preparation from the moment the stimulus is presented until speech starts. This method makes it possible to examine the undergoing planning processes at different moments before speech onset (see Griffin, 2004; Meyer, 2004, for reviews of the uses of eye-tracking methodology in language production).

**Figure 1:**
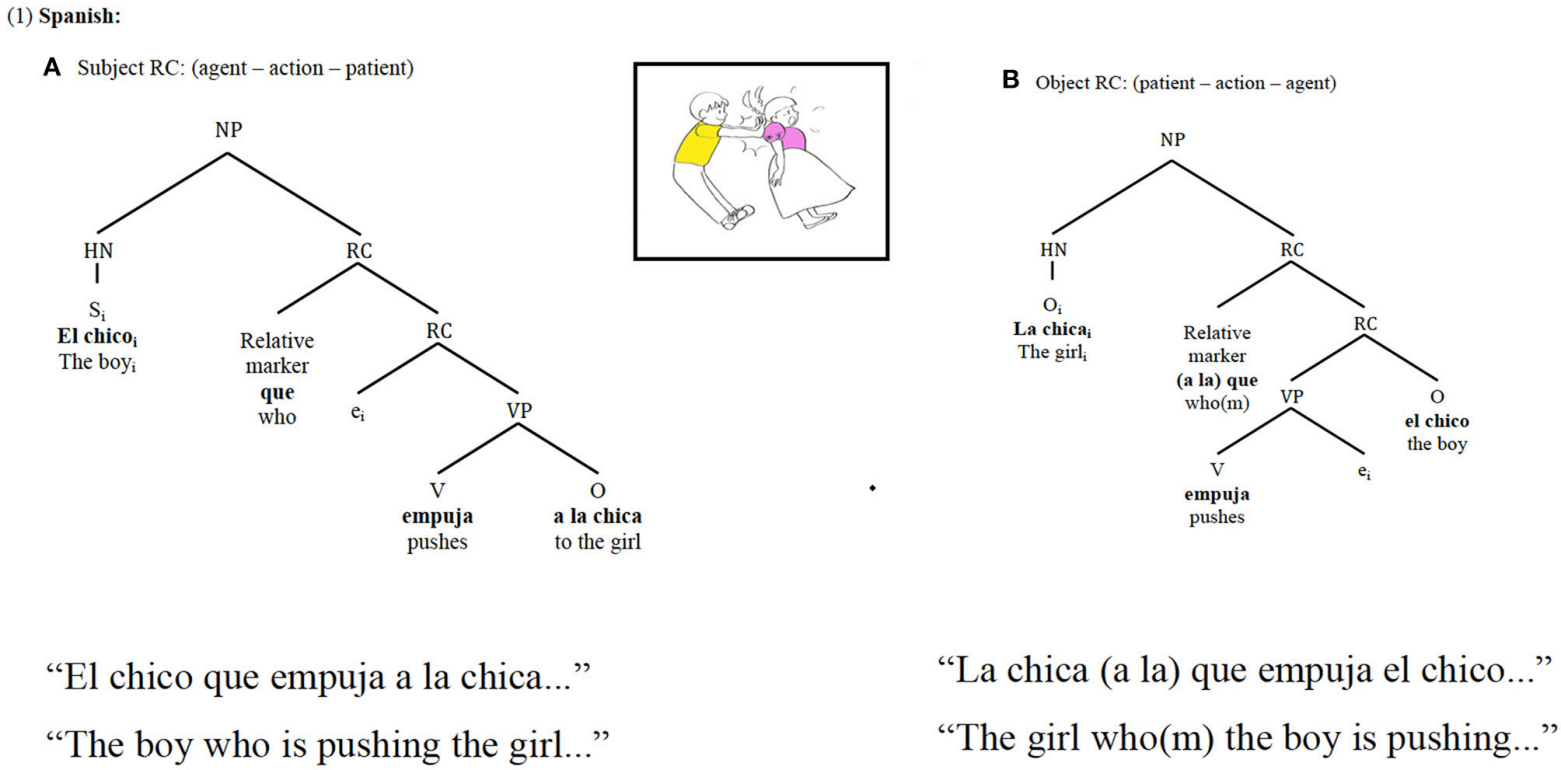
Spanish subject RCs **(A)** and object RCs **(B)** syntactic and thematic role relations and constituent order.

Spanish RCs are head-initial structures; that is, when producing an RC in Spanish, the head noun (hereafter HN) will be uttered first, regardless of its grammatical function within the subordinate clause. Nonetheless, Spanish RCs allow for greater word order flexibility inside the subordinate clause: in object RCs (with an active verb), the subject may follow the verb, which is the preferred order, thus resulting in no differences in word order between subject and object RCs. As can be seen in Figure 1, the order HN—verb—subordinate NP is kept constant across subject and object-RCs.

**Figure 2:**
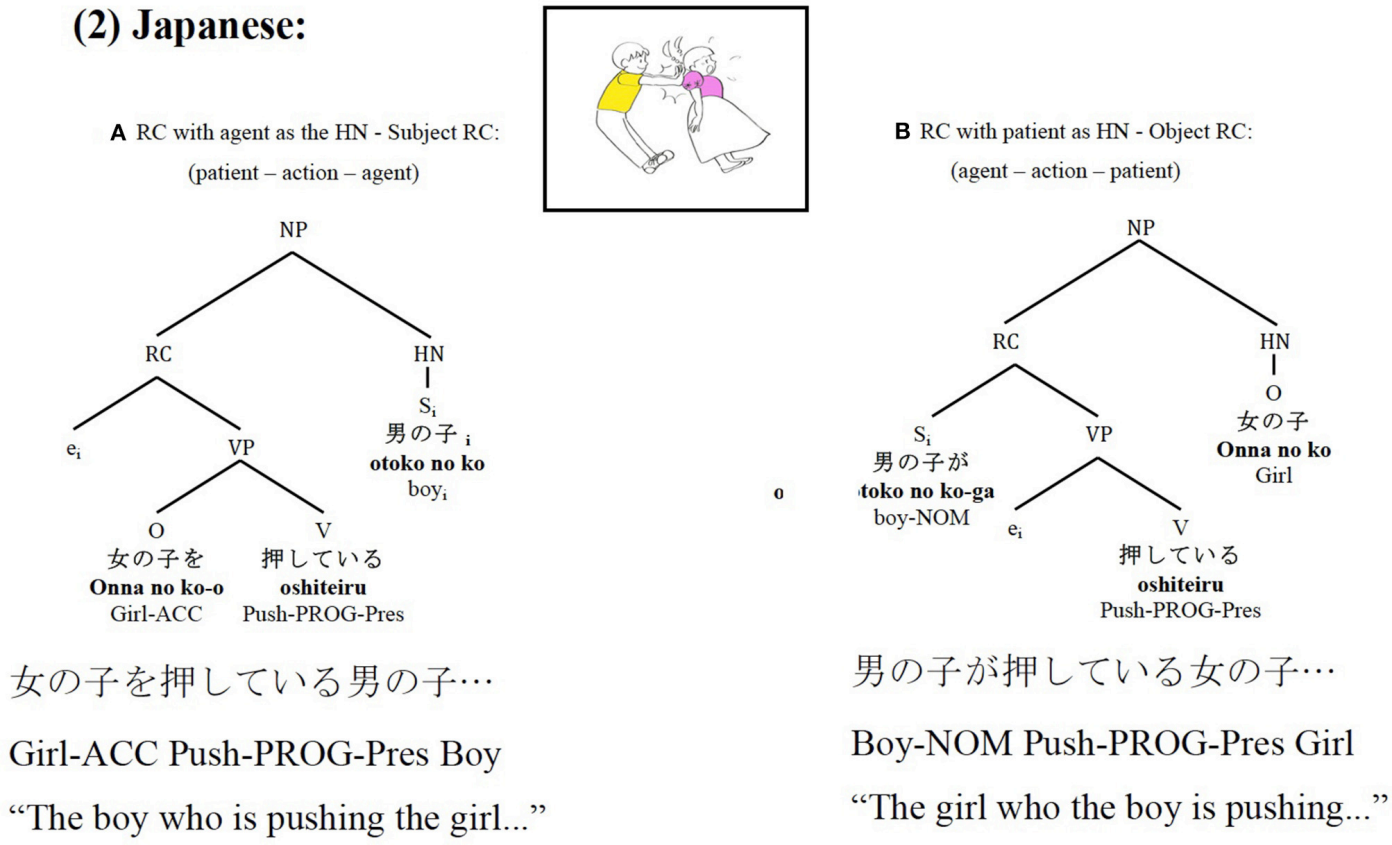
Japanese subject RCs **(A)** and object RCs **(B)** syntactic and thematic role relations and constituent order.

In contrast, Japanese, a head-final language, places the HN of the RC after the subordinate clause^3^. Thus, interestingly, in Japanese the syntactically highest constituent of the RC is not the first placed element, but the last one, as can be seen in Figure 2, which yields the opposite word order than in Spanish.

Yet, despite this difference, in both Japanese (Montag and MacDonald, 2009) and Spanish RCs (Gennari et al., 2012), animate elements (that is, conceptually salient elements) are equally assigned a more prominent syntactic position. In both cases, animate HNs are assigned the subject function more often.

Thus, the comparison of the same structure in two languages with opposite word orders will allow to figure out whether the prioritized information when undertaking syntactic planning is relational or non-relational; in other words, whether incrementality is hierarchically or linearly driven.

Due to its HN-initial order, Spanish yields a pattern in which it is difficult to tell apart the most dominant position from the first uttered element. On the contrary, Japanese (HN-final) allows us to identify the kind of information that is prioritized in order to undertake linguistic encoding: either relational, where the construction of an overall structure takes priority, or non-relational, where the retrieval of lexical items as they are going to be uttered determines the choice of sentence structure.

If linear incrementality were prioritized, with stronger reliance on non-relational information, we would expect to find a pattern in which the items are fixated in the same order as they are uttered. Critically, we would expect this to be the case for both Spanish and Japanese, regardless of the position of the HN. As a consequence, the order of gazes would be reversed between Japanese and Spanish in subject and object RCs.

On the other hand, if speech planning favors hierarchical incrementality, thus prioritizing relational information, we would expect a pattern in which, following apprehension of the event, the structure is planned before name-related gazes take place. This pattern would be visible in both in Spanish and Japanese. Importantly, in contrast with simple transitive clauses, where planning of the structure results in convergent gazes between agent and patient (Konopka and Meyer, 2014), we would expect that the construction of the structure in RCs would produce a pattern of increased gazes to the HN, as the most dominant element, on which the relative clause is dependent. As a result, if linguistic encoding is guided hierarchically, participants should focus on the HN around 400 ms. after picture onset, and these gazes would last until lexical retrieval starts. From that moment, Spanish speakers will keep focusing the HN, as the first uttered element, while Japanese speakers are expected to switch their gazes, turning to the first uttered element before speech onset occurs. A summary of predictions is shown in Table 1,^4^.

**Table 1:** Summary of expected differences in order of gazes from 400 ms. onwards, before name-related gazes start: Constant grammatical function and different word order across languages.

	**Agent as HN**	**Patient as HN**
If linear incrementality takes place	Japanese ≠ Spanish	Japanese ≠ Spanish
If hierarchical incrementality takes place	Japanese = Spanish	Japanese = Spanish

Conceptual accessibility, in this case guided by animacy, might play a role in modulating the access to non-relational information (by means of individual saliency) or to relational information (which is related to the prototypicality of the whole scene). Thus, in addition, we analyzed the role that animacy plays in linguistic planning, that is, whether it exerts an influence in RC production after apprehension has taken place, once linguistic encoding has started. We expect that if animacy has an influence on early linguistic planning, animate HNs will be focused more prominently than inanimate HNs from the very beginning, thus showing encoding preferences. On the other hand, if animacy plays no role in early linguistic encoding processes, we should find no differences between animate and inanimate HNs, in both RCs with agent and with patient as the HNs. It should be borne in mind that any possible effect of animacy will be reflected on the pattern of gazes to the participants involved in the scene, not in their order of mention. (The reader is referred to Tables 2 and 3 below, for examples of correct responses in the production task).

**Table 2:** Examples of critical items in the three animacy conditions with provided questions and examples of accepted responses in Spanish.

**Example of picture**	**Provided questions in Spanish**	**Examples of accepted responses**
Animate agent—Animate patient (AA) 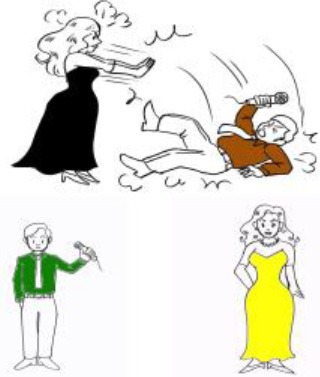	1. Agent-HN: ¿Quién lleva un vestido negro? *Lit:* “Who wears a dress black?” (“Who is wearing a black dress?”)	1. Agent-HN: - La actriz que derriba a un periodista (lleva un vestido negro) *Lit:* “The actress who knocks down the journalist (wears a dress black)” (“The actress who knocks down the journalist (is wearing a black dress)”)
	2. Patient-HN: ¿Quién lleva una camisa marrón? *Lit:* “Who wears a shirt brown?” (“Who is wearing a brown shirt?”)	2. Patient-HN: - *Active voice:* El periodista al que derriba la actriz (lleva una camisa marrón) *Lit:* “The journalist whom knocks down the actress (wears a shirt brown)” (“The journalist whom the actress knocks down (is wearing a brown shirt)”)
	*Provided verb:* “Derribar” (“Knock down”)	- *Passive voice:* El periodista que está siendo derribado por la actriz (lleva una camisa marrón) *Lit:* The journalist whom is being knocked down by the actress (wears a shirt brown) (“The journalist whom is being knocked down by the actress (is wearing a brown shirt)”)
Animate agent—Inanimate patient (AI) 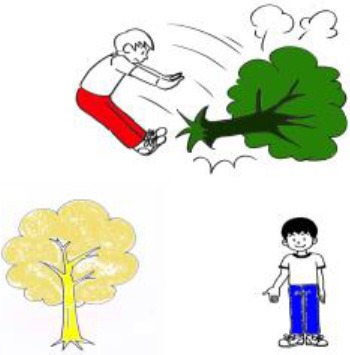	1. Agent-HN: ¿Quién lleva unos pantalones rojos? *Lit:* “Who wears trousers red?” (“Who is wearing red trousers?”)	1. Agent-HN: - El chico que derriba un árbol (lleva unos pantalones rojos) *Lit:* “The boy who knocks down the tree (wears trousers red)” (“The boy who knocks down the tree (is wearing red trousers)”)
	2. Patient-HN: ¿Qué es verde? (“What is green?”)	2. Patient-HN: - *Active voice:* El árbol que derriba el chico (es verde) *Lit:* “The tree that knocks down the boy (is green)” (“The tree that the boy is knocking down (is green)”)
	*Provided verb:* “Derribar” (“Knock down”)	- *Passive voice:* El árbol que está siendo derribado por el chico (es verde) (“The tree that is being knocked down by the boy (is green)”)
Inanimate agent—Animate patient (IA) 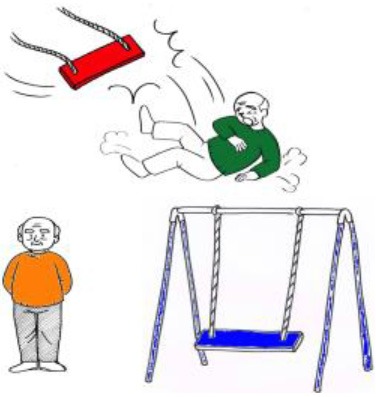	1. Agent-HN: ¿Qué es rojo? (“What is red?”)	1. Agent-HN: - El columpio que derriba a un anciano (es rojo) (“The swing that knocks down the old man (is red)”)
	2. Patient-HN: ¿Quién lleva un jersey verde? *Lit:* “Who wears a sweater green?” (“Who is wearing a green sweater?”)	2. Patient-HN: - *Active voice:* El anciano al que derriba el columpio (lleva un jersey verde) *Lit:* “The old man whom knocks down the swing (wears a sweater green)” (“The old man whom the swing knocks down (is wearing a green sweater)”)
	*Provided verb:* “Derribar” (“Knock down”)	- *Passive voice:* El anciano que está siendo derribado por el columpio (lleva un jersey verde) *Lit:* “The old man that is being knocked down by the swing (wears a sweater green)” (“The old man that is being knocked down by the swing (is wearing a green sweater)”)

**Table 3:** Examples of critical items in the three animacy conditions with provided questions and examples of accepted responses in Japanese.

**Example of picture**	**Provided questions in Japanese**	**Examples of accepted responses**
Animate agent—Animate patient (AA) 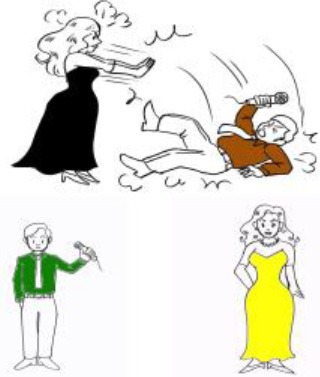	1. Agent-HN: 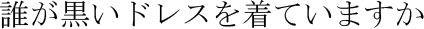 ? Who-NOM black dress-ACC is wearing-Q? (“Who is wearing a black dress?”)	1. Agent-HN: - 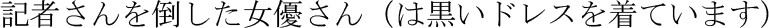 Journalist-ACC knocked down actress (-TOP black dress-ACC is wearing) (“The actress who knocks down the journalist (is wearing a black dress)”)
	2. Patient-HN: 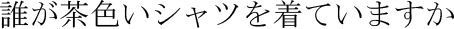 Who-NOM brown shirt-ACC is wearing-Q? (“Who is wearing a brown shirt?”)	2. Patient-HN: - *Active voice:* 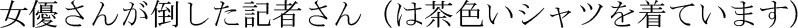 Actress-NOM knocked down journalist (-TOP brown shirt-ACC is wearing) (“The journalist whom the actress knocks down (is wearing a brown shirt)”)
	*Provided verb:* 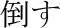 (“Knock down”)	- *Passive voice:* 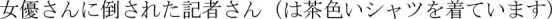 Actress-DAT is knocked down journalist (-TOP brown shirt-ACC is wearing) (“The journalist whom is being knocked down by the actress (is wearing a brown shirt)”)
Animate agent—Inanimate patient (AI) 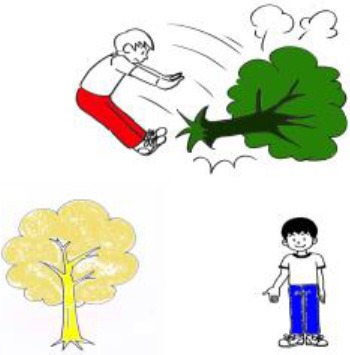	1. Agent-HN: 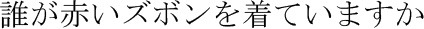 Who-NOM red trousers-ACC is wearing-Q? (“Who is wearing red trousers?”)	1. Agent-HN: - 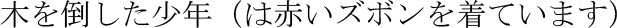 Tree-ACC knocked down boy (-TOP red trousers-ACC is wearing) (“The boy who knocks down the tree (is wearing red trousers)”)
	2. Patient-HN: 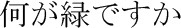 What-NOM green is-Q? (“What is green?”)	2. Patient-HN: - *Active voice:* 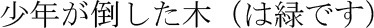 ? Boy-NOM knocked down tree (-TOP green is) (“The tree that the boy is knocking down (is green)”)
	*Provided verb:* 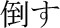 (“Knock down”)	- *Passive voice:* 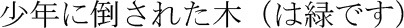 Boy-DAT is knocked down tree (-TOP green is) (“The tree that is being knocked down by the boy (is green)”)
Inanimate agent—Animate patient (IA) 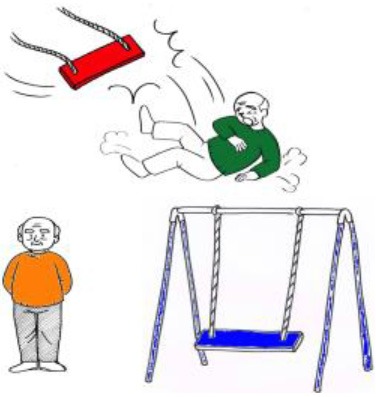	1. Agent-HN: 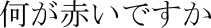 ? What-NOM red is-Q? (“What is red?”)	1. Agent-HN: - 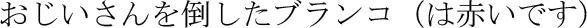 Old man-ACC knocked down swing (-TOP red is) (“The swing that knocks down the old man (is red)”)
	2. Patient-HN: 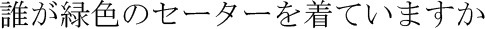 ? Who-NOM green sweater-ACC is wearing-Q? (“Who is wearing a green sweater?”)	2. Patient-HN: - *Active voice:* 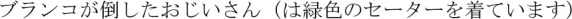 Swing-NOM knocked down old man (-TOP green sweater-ACC is wearing) (“The old man whom the swing knocks down (is wearing a green sweater)”)
	*Provided verb:* 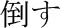 (“Knock down”)	- *Passive voice:* 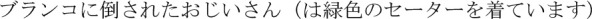 Swing-DAT is knocked down old man (-TOP green sweater-ACC is wearing) (“The old man that is being knocked down by the swing (is wearing a green sweater)”)

In what follows, we present two experiments intended to clarify the time-course of the production of RCs, focusing on the information that is prioritized after apprehension, when linguistic encoding starts. We used the eye-tracking methodology, monitoring participants' eye movements before and during the production of RCs in a visual-world paradigm (see Griffin, 2004 for a review on the uses of the eye-tracking methodology in language production). In the first experiment, we report data from Spanish. The second experiment was conducted in Japanese, using the same method and procedure. In the final discussion, a comparison between both experiments will be carried out. The comparison of both experiments will help us understand the role of word order differences and conceptual saliency after scene apprehension is over.

## Experiment 1: relative clause production in Spanish

### Method

#### Participants

Thirty-one Castilian Spanish native speakers, undergraduate or graduate students at the Universidad Autónoma de Madrid (mean age: 22.7) participated in this study. All participants reported normal or corrected-to-normal vision.

#### Materials and design

Thirty critical plus thirty filler colored pictures were presented for description. Each critical picture depicted four participants, either all animate (human) or two animate and the other two inanimate. Two of the participants were involved in a transitive action, while the other two remained inactive, acting as contrastive elements. Examples of critical pictures can be seen in Table 2,^5^. Position of the four elements was counterbalanced in the up-down and left-right axes. There were ten different actions, each coupled with a different animacy condition.

Fillers consisted of a four-participant scene showing intransitive actions or objects with contrasting sizes. As can be seen in Table 2, items were presented twice during the task, with questions referring to either the agent or the patient of the scene.

A 3 × 2 factorial design was used with two within-participant independent variables, namely, (1) “animacy distribution,” with three levels: Animate agent—Animate patient (AA), Animate agent—Inanimate patient (AI), and Inanimate agent—Animate patient (IA); and (2) “head noun (HN) of the relative clause” (henceforward, RC-Head Noun), with two levels: Agent-HN and Patient-HN.

Along the experiment, each participant saw a list of 120 pictures (30 critical and 30 fillers repeated twice), each preceded by a written question and a verb. Within the critical items, there were 10 pictures of each animacy distribution (i.e., AA, AI, and IA). The list of items was divided into two blocks, each with 60 items (30 critical and 30 fillers). Within each block, each of the critical pictures followed a question referring to one of the participants in the event (the agent or the patient), such that 15 of the critical items in each block were intended to have the agent as HN, and the other 15 the patient as HN, with the HN reversed for each item in the other block. Thus, the participants saw the same critical items twice, but with a different antecedent HN on each block. All 60 items in each block were randomized.

#### Apparatus

A Tobii T120 eye-tracker with a sampling rate of 60 Hz was used. Stimuli presentation and data collection were performed using Tobii Studio 2.0. Responses were recorded and transcribed for analysis.

#### Procedure

Participants were tested individually. Before the task, each participant was asked to separately identify all of the animate and inanimate participants as well as the main actions that would be presented during the task. If the participant was unable to identify the element, the experimenter provided the correct answer. This was done to ensure participants understood all of the elements involved in the scenes and the roles represented therein. They were encouraged to respond with the most natural description during the task.

**Figure 3:**
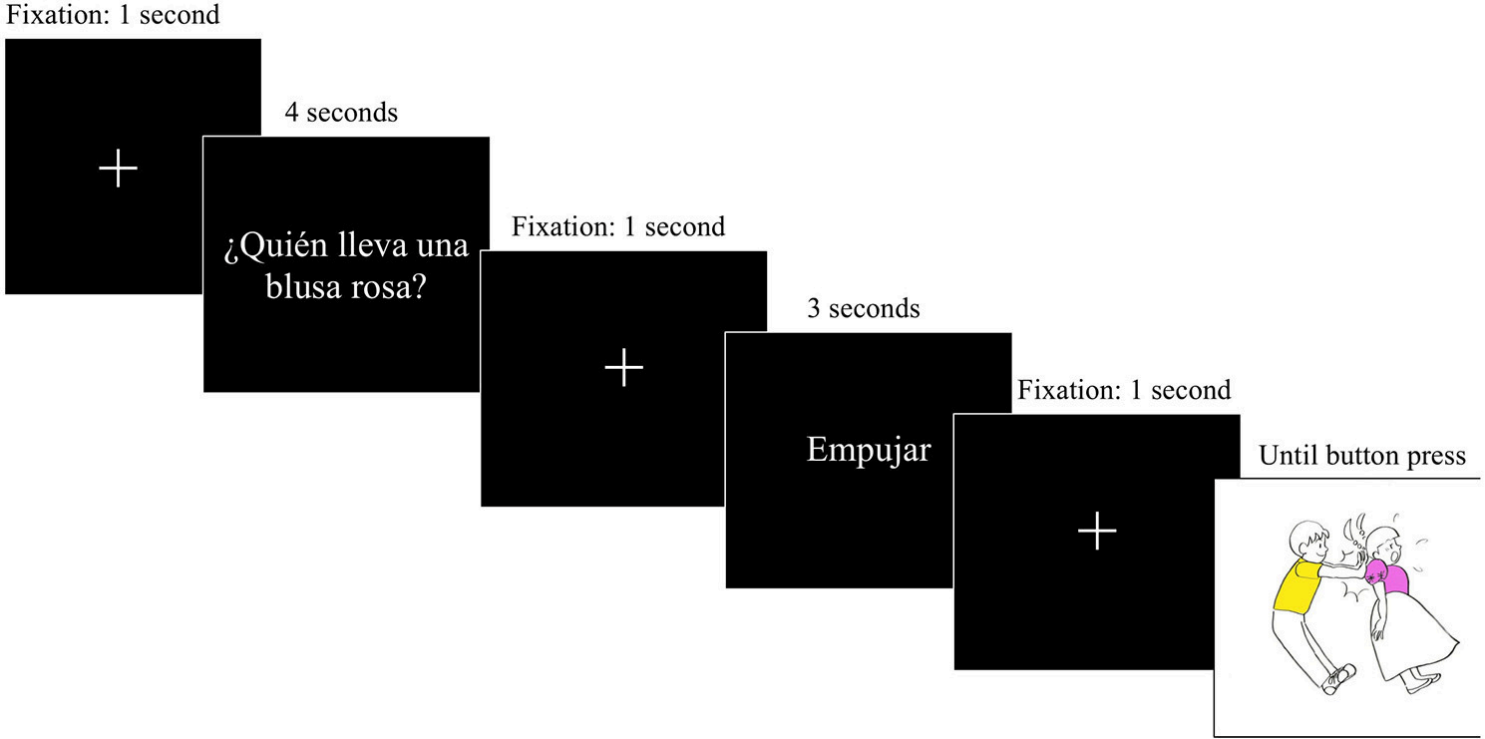
Representation of each trial during the experiment.

After this familiarization stage, the experimental task began. First, the task was explained to each participant and a built-in 9-point calibration was conducted. There were four practice items before the task. The task consisted of answering a question presented on the screen referring to the agent or the patient of the event. These questions asked about the color or shape of one of the participants involved in the transitive action of the picture (e.g., “¿Quién lleva una blusa rosa?,” “Who is wearing a pink blouse?”). After that, a verb in the infinitive form appeared (e.g., “Empujar”—“To push”). Participants were instructed to use this verb in their answers. Finally, the picture appeared on the screen. At this point, the participant had to answer the question while using the provided verb. When they finished the sentence, they pressed the space key in order to move forward to the next item. The sequence of events on each trial can be seen in Figure 3 below.

There was no time limit, but participants were encouraged to answer with the first and most natural response that came to mind. There was a 5–10 min break between the two blocks of the experiment. The complete session, including the calibration of the eyetracker and the practice items, lasted about 60 min.

### Results

#### Data analysis

Uttered responses were recorded and transcribed for analysis. The following types of responses were excluded from gaze analysis: (a) responses that did not include an RC or begin with one, (b) responses that differed in meaning with respect to the verb provided or picked the wrong HN, (c) responses that failed to overtly mention the two participants involved in the event (e.g., sentences with agent dropped), and (d) active object RCs in which the subject preceded the verb (e.g., sentences like “el chico al que la chica empuja”—*The boy whom the girl pushes*, unlike “el chico al que empuja la chica”—*The boy whom pushes the girl*, which happen to be much more frequent in Spanish). This last type of response was excluded so as to keep constant the effect of the verb on fixation patterns^6^. Examples of possible correct responses can be found in Table 2 above^7^. Additionally, Speech Onset (SO) was measured manually with Praat (version 5.3.71) (Boersma and Weenink, 2014). Subsequently, the average and standard deviation of each participant was calculated. Speech latencies greater than 10 s or more than two standard deviations from each participant's average SO were removed as well. As a result, three participants were excluded, since they did not provide any valid response. Excluded responses under these criteria amounted to 44.87 % of all responses (31.91% of RC responses), with a sample of 28 valid participants, and a total of 1005 responses to analyze^8^. Responses were classified as active, passive or impersonal sentences. The “impersonal sentences” category contains both impersonal and reflexive sentences^9^.

Subsequently, we analyzed fixations for a time period from picture onset up to 4,500 ms. (so as to include the period up to speech onset times in Japanese, which were slower in our study). Interest areas were defined for both agent and patient. Accordingly, proportions of fixations to agent and patient were measured. Gaze position was recorded every 16.67 ms; as such, data points were grouped into 50 ms. bins in order to capture the time-course of gazes from picture onset onwards. This information is shown in the figures below. Finally, time windows (TW) larger than 50 ms were defined and used for statistical comparisons. Thus, we defined and statistically compared the following TWs, based on the previous literature and our own data: TW1: 1–350 ms. [average for apprehension period in previous literature (e.g., Griffin and Bock, 2000)], TW2: 400–1,000 ms. (average for naming latency of single lexical items Cuetos et al., 1999; Nishimoto et al., 2005,^10^, TW3: 1,050–2,500 ms. (speech onset average for Spanish in our data) and TW4: 2,550–4,500 ms. (speech onset average for Japanese in our data).

We created three linear mixed-effects models: one for participant responses, with the proportion of passive sentences as the dependent variable; and two for gaze patterns in each TW, with proportion of looks to either the agent (in one model) or patient (in the other) as dependent variables. For all models, RC-Head Noun, Animacy, and their interaction were included as fixed effects and as crossed-random slopes (or, when the model was not improved by their inclusion, as subject and item intercepts).

#### Results

**Figure 4:**
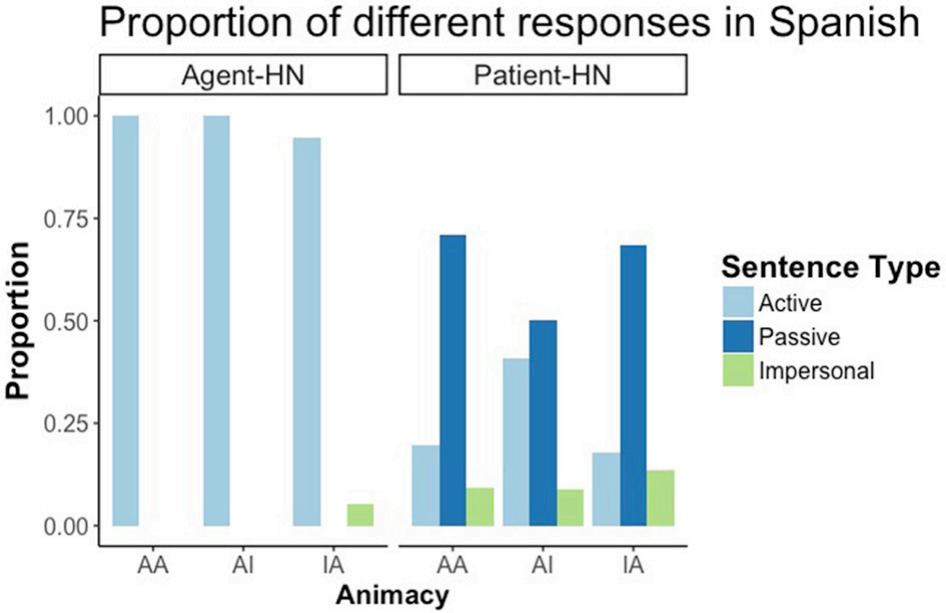
Proportion of active, passive and impersonal responses in RCs with the Agent as HN and the Patient as HN in Spanish.

Regarding the form of participants' responses, we found a pattern that replicated previous findings in the literature (i.e., Gennari et al., 2012). When the patient was the HN of the sentence, the response was mediated by the animacy of the elements. There was a main effect of both RC-Head Noun (Agent-HN vs Patient-HN) (*p* < 0.001) and animacy. While RCs with the agent as HN gave rise to active sentences neccessarily, RCs with the patient as HN showed a wider variety of responses dependent on animacy distribution, thus yielding an interaction between both factors. Regarding animacy, AA, and IA sentences did not differ from each other, both producing a greater number of passive sentences than items with AI (*p* < 0.001). That is, animate patients (i.e., animate HNs) were more prone to be promoted and produced as subjects than inanimate ones, resulting in passive RCs (see Figure 4).

**Figure 5:**
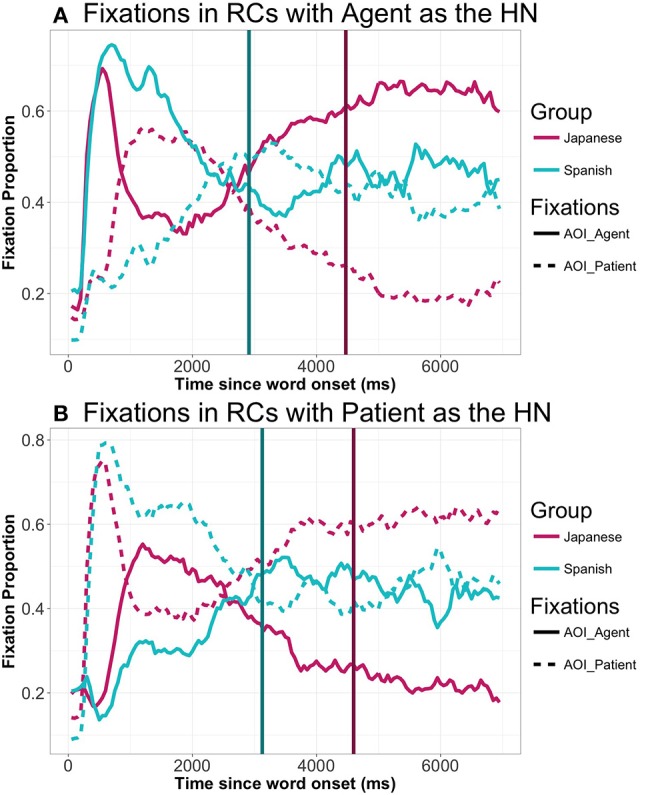
General gaze patterns to agent and patient in Spanish and Japanese RCs with the agent as HN **(A)** and with the patient as HN **(B)** (all animacy combinations collapsed): From picture onset until 7,000 ms. Vertical lines represents speech onset in either language.

After this, we analyzed the gaze patterns in the different types of experimental sentences. The observed pattern was one in which participants tend to look at the element they are going to mention first in the sentence before speech onset. At this point, they stop looking at it before articulating it, so as to shift to the next element (see Figure 5 below^11^). The average SO was 2,984 ms., with the Agent-HN sentences having faster SO's than those with the Patient as HN (Agent-HN: 2913 ms., Patient-HN: 3127 ms., *t* = −2.769, *p* = 0.007).

In the period corresponding to the first TW (0-350 ms after picture onset), the apprehension period, we found no differences due to RC-Head noun type in either gazes to the agent (*t* = 0.96, *p* = 0.35) or to the patient (*t* = −1.54, *p* = 0.13). Similarly, during this period, there are no significant differences due to the animacy of the elements in gazes to the agent (*t* = 0.67, *p* = 0.51), and only marginally significant in gazes to the patient (*t* = 1.91, *p* = 0.063), with the IA condition being numerically higher than the other two conditions.

**Figure 6:**
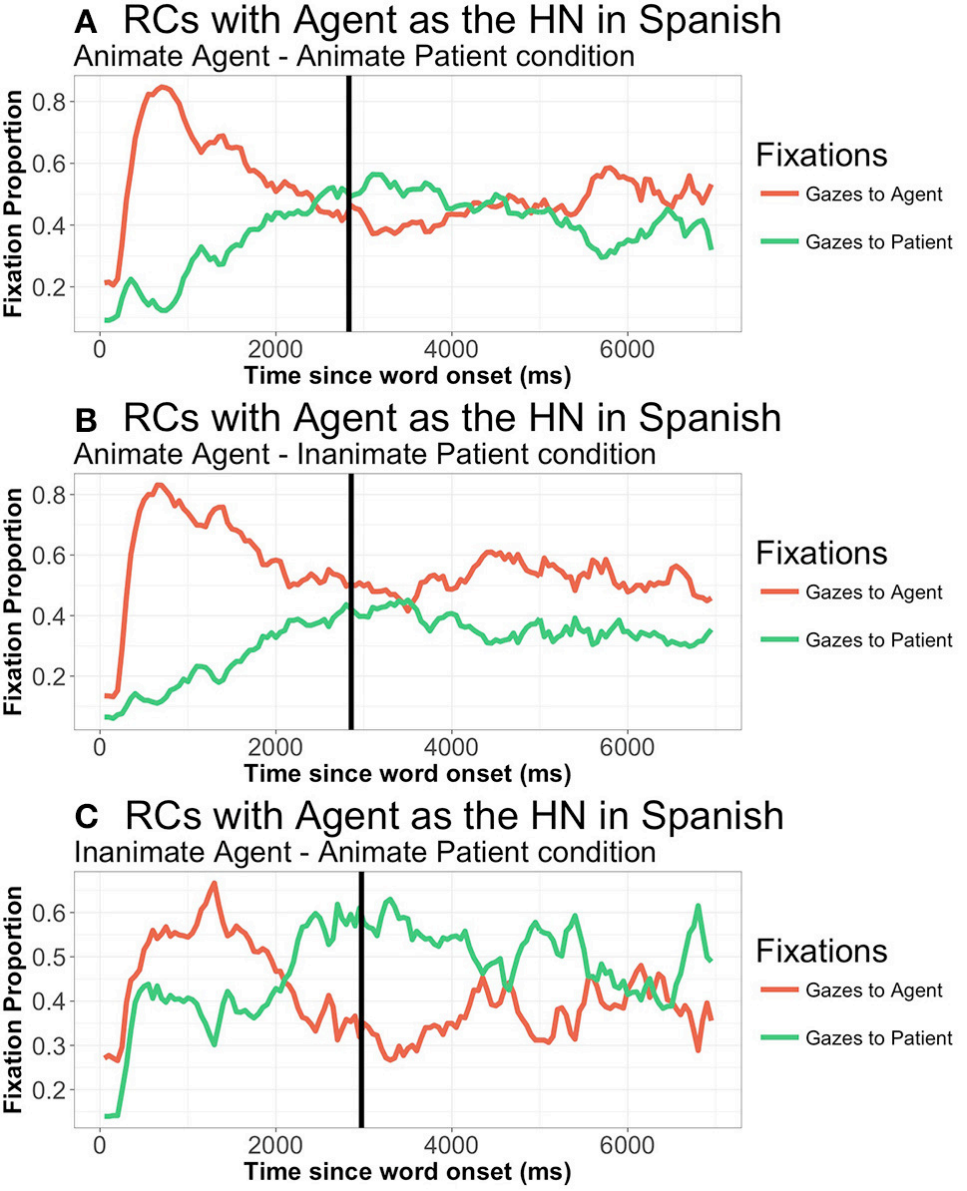
Patterns of gazes to agent and patient in Spanish RCs when the agent was the HN, from picture onset until 7,000 ms., in all three animacy combinations: **(A)** Animate agent—Animate patient; **(B)** Animate agent—Inanimate patient; **(C)** Inanimate Agent—Animate patient. Similar pattern when patient was the HN, figures omitted. Vertical line represents speech onset.

From 400 to 2,500 ms. (TWs 2 and 3), gaze patterns to both agent and patient differed between RC Head noun types (all differences *p* < 0.001), with gazes mostly directed to the HN / first element of the RC. In contrast, there were no significant differences due to RC Head noun type from that moment onwards, that is, after speech onset (from TW4 onwards). Regarding the effect of animacy, there is a significant main effect of animacy from 400 to 1,000 ms. (TW2) for gazes to the agent (*t* = −2.76, *p* = 0.009) but not for gazes to the patient. Participants focus more extensively on animate agents than inanimate ones, and more so when the patient is inanimate (AI = 0.44141, AA = 0.36121, IA = 0.26601). This pattern remains in the following TWs, although it is only marginally significant in all of them (all TWs, *p* < 0.09) (see Figure 6).

The results of Experiment 1 exhibit a pattern in which speakers focus on the first and syntactically most dominant element from 400 ms. and keep focusing on it until speech onset. Animacy effects do not show up during the apprehension stage, but only after linguistic encoding has started (that is, from TW2 onwards). These effects, however, might be due to lexical access during the latter. A comparison with Japanese will allow us to further explore this pattern and analyze any possible cross-linguistic differences or similarities.

## Experiment 2: relative clause production in Japanese

### Method

#### Participants

Thirty-two Japanese native speakers, undergraduate or graduate students at Hiroshima University (mean age: 20, range: 18–23) participated in this study. One participant was excluded, since only 6.5 % of his gazes were recorded. All participants reported normal or corrected-to-normal vision.

#### Materials, design, apparatus and procedure

All were exactly the same as in Experiment 1, except that instructions, questions and the verbs and other lexical items were translated into Japanese. Examples of the provided questions and accepted answers in Japanese can be seen in Table 3.

### Results

#### Data analysis

The same analysis and Time Windows (TWs) were used as in Experiment 1. We excluded from gaze analysis the following types of responses: (a) responses that did not start with the relative clause; (b) responses in which the HN was uttered before the relative clause; (c) responses that failed to overtly mention the two participants involved in the event (e.g., sentences with agent dropped); and (d) responses in which speech latencies were greater than 10 s or more than two standard deviations from each participant's average SO. Examples of possible correct responses can be found in Table 3 above. Excluded responses amounted to 33.28 % of all responses (27.96% of RC responses)^12^, with a sample of 31 valid participants, and a total of 1319 responses to analyze.

#### Results

**Figure 7:**
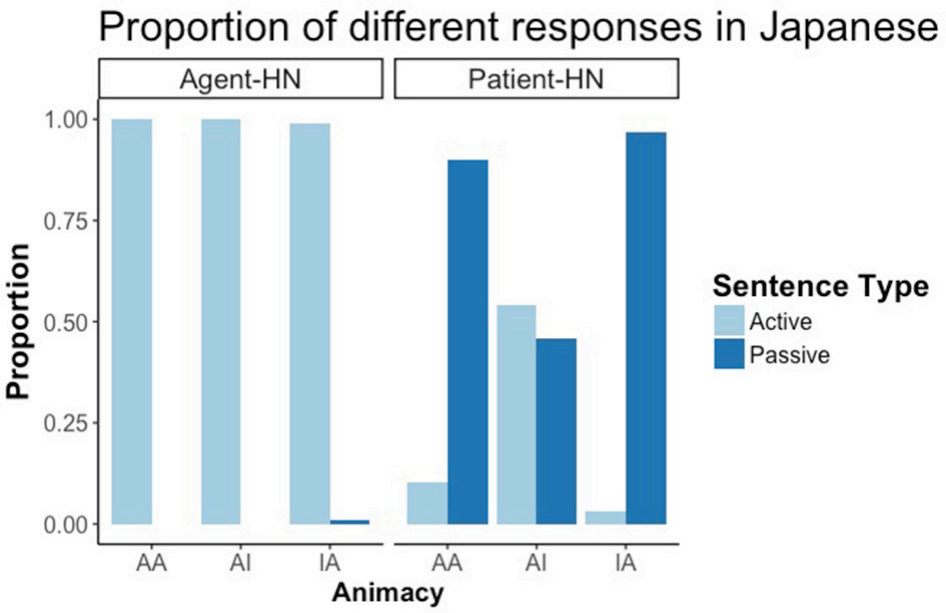
Proportion of active, passive and impersonal responses in RCs with the Agent as HN and the Patient as HN in Japanese.

Similarly to Experiment 1, and in accordance with previous literature in Japanese RCs (Montag and MacDonald, 2009), the voice of the RCs with the patient as HN depended on the animacy of the HN. There was a main effect of RC Head noun type and of animacy, as well as an interaction between both factors (all differences *p* < 0.01). Participants produced necessarily active RCs when Agent was the HN, while the proportion of active and passive RCs when patient was the HN depended on animacy, Participants produced a higher proportion of passive sentences when the HN was animate than when it was inanimate (with no differences between AA and IA conditions), thus promoting animate HNs to the subject function despite its final position (Figure 7).

Next, we examined the proportion of fixations across the different types of sentences. A general examination of the proportion of gazes along the time-course shows a pattern in which speakers focus on the HN initially, before shifting their gazes to the element that they are going to produce first (the subordinate NP) (Figure 5 above). SO was slower in Japanese than in Spanish, with a mean of 4,534 ms. Thus, compared to Spanish, in Japanese there is a delay in starting the utterance that stretches a whole time window (approximately 2,000 ms.).

A closer look at gaze patterns revealed an effect of RC-Head noun type in both gazes to the agent (*t* = 4.44, *p* < 0.001) and to the patient (*t* = −3.61, *p* < 0.001) in the first TW (0 to 350 ms.). Participants start to focus their gazes on the element that is going to be the HN from 300 ms. onwards when the HN is the agent: no differences due to RC-Head noun type were found in the 0 to 300 ms. period: *t* = 1.348, *p* = 0.204. Differences start as early as 250 ms. in the case of gazes to the patient (again no differences from 0 to 250 ms.: t = −0.890, *p* = 0.3846). From that moment onwards, there was a main effect of RC-Head noun type in all analyzed TWs, with different patterns depending on the TW. Thus, from 400 ms. to 1,000 ms. (TW2), the HN is fixated to a greater extent, even though it is not placed at initial position (agent: Agent-HN = 0.56, Patient-HN = 0.33, *t* = 6.47, *p* < 0.001; patient: Agent-HN = 0.36, Patient-HN = 0.60, *t* = −6.12, *p* < 0.001)^13^. However, the crossing point at which the overall pattern of proportion of gazes to agent and patient changes is located at 850 ms. in RCs with the Agent as HN (proportion of gazes to the agent: 0.48; proportion of gazes to the patient 0.46) and at 900 ms. in RCs with the Patient as HN (proportion of gazes to the agent: 0.47; proportion of gazes to the patient 0.46). From 1,050 to 2,500 ms. (TW 3), participants shift their gazes to the element that is placed at sentence-initial position (i.e., the subordinate NP) (both gazes to agent and patient: *ps* < 0.05). After this brief focus on the first element, participants shift again to the HN, which is focused extensively even after SO (TW 4): gazes to both agent and patient: *ps* < 0.001. This pattern suggests that participants tentatively prepare the utterance by focusing on the HN before starting lexical retrieval. The tendency to go back to the HN quite early (despite it being the second uttered element) shows a strong reliance on the HN when planning RCs.

**Figure 8:**
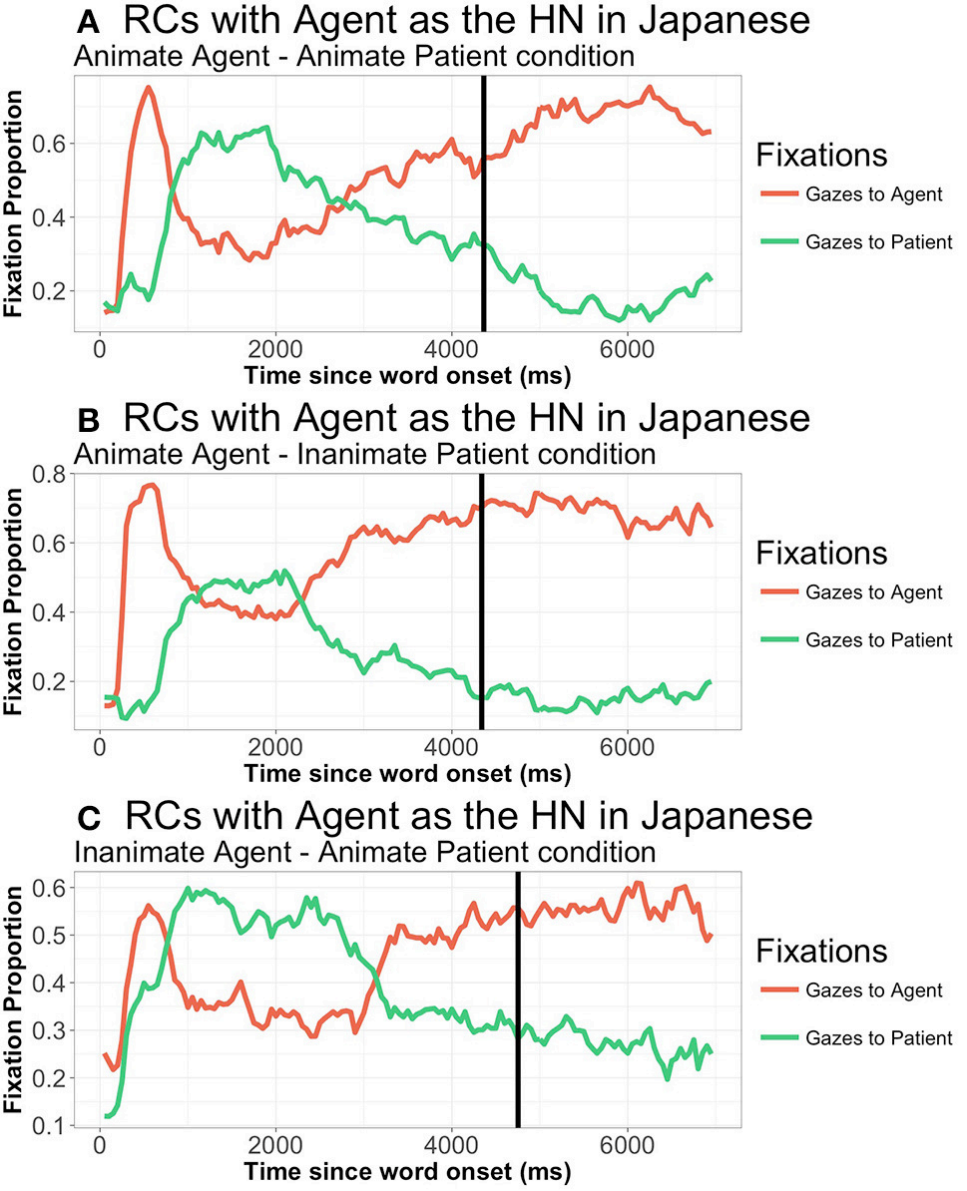
Patterns of gazes to agent and patient in Japanese RCs when the agent was the HN, from picture onset until 7,000 ms., in all three animacy combinations: **(A)** Animate agent—Animate patient; **(B)** Animate agent—Inanimate patient; **(C)** Inanimate agent—Animate patient. Similar pattern when patient was the HN, figures omitted. Vertical line represents speech onset.

Regarding the effect of animacy (Figure 8), there is a marginal effect of animacy from 0 to 300 ms.) on gazes to the agent (*t* = 1.977; *p* = 0.056). Interestingly, participants look more extensively at the agent in IA conditions, that is, they focus on the *inanimate* doer of the action. TW2 and TW3 show no effects of animacy, while the last analyzed TW (from 2,550 to 4,500 ms.) again shows a marginal effect in fixations to the agent, in this case with more fixations to the animate agent (HN) in AI conditions. There is no effect of animacy on fixations to the patient in any of the analyzed TWs.

**Figure 9:**
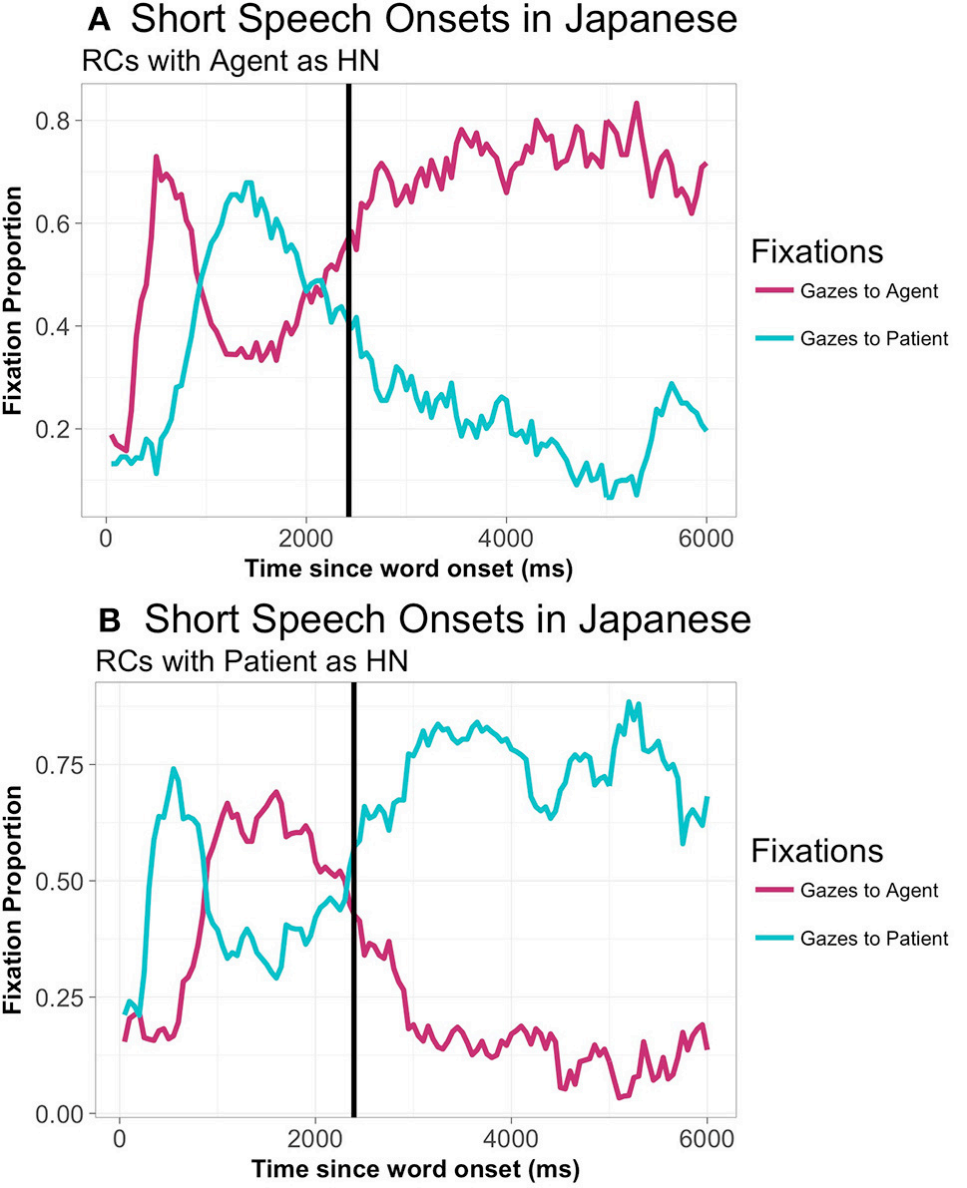
Gaze patterns to agent and patient in Japanese RCs with the agent as HN **(A)** and with the patient as HN **(B)** (all animacy combinations collapsed), only in responses with speech onsets from 1,000 ms to 3,000 ms.: From picture onset until 7,000 ms. The vertical line represents the average SO.

Since speech onset took place later in Japanese than in Spanish, there arises the possibility that the pattern found in Japanese was due to this delay. In order to check whether the pattern found was due to long speech onset latencies, we additionally analyzed the gaze patterns of items with speech onsets between 1,000 and 3,000 ms. There were a total of 614 utterances (47.31% of the total of included utterances), coming from 26 participants, distributed across the 60 experimental items. Within this subset, the SO mean was 2,328 ms. (agent-HN: 2311 ms.; patient-HN: 2,346 ms.). As can be seen in Figure 9, the general pattern did not change when taking into account only those speech onset latencies similar to Spanish: participants focus on the HN from 300 to 850 ms. on average, before turning their gazes to the first uttered noun, which is not the one that is focused first.

In summary, Japanese speakers show a gaze pattern that does not follow the linear order of the utterance. Rather, there is a period of about 900 ms. in which the HN, the last placed element, is extensively fixated. These results support the idea of a structural planning of the RC before lexical retrieval starts, with participants largely focusing on the syntactically most dominant element before engaging in the search for words that will form the final utterance. The lack of effects of animacy is also remarkable, with participants engaging in a planning process that is not mediated by the saliency of isolated elements, but rather by the general prototypicality of the scene.

## General discussion

In this paper, we have reported two experiments comparing the planning processes involved in RC production in a head initial language and a head final language from an online perspective. We focused on the information that is prioritized in linguistic encoding, either structural or lexical, after conceptual apprehension occurs and gives way to linguistic encoding.

In order to explore this issue, we compared the production planning of RCs in Spanish (experiment 1) and Japanese (experiment 2). RCs exhibit the opposite word order in Spanish (head-initial language) and Japanese (head-final language), the latter having a structure in which the HN is postponed to its subordinate constituents within the relative clause. Additionally, we controlled for the relative salience of each of the elements involved by manipulating animacy, a variable that has been reported as having an effect in the choice of grammatical function either regardless of, or in consonance with, word order.

Despite the cross-linguistic difference in word order, gaze patterns from 0 to 350 ms. and from 400 ms. to 1050 ms. were quite similar in both languages. Thus, there is a brief period of around 350 ms. in which neither agent or patient are preferentially focused. This period is slightly shorter in Japanese, with participants focusing preferentially on the HN from 300 ms. onwards. We consider this period to correspond to the apprehension stage, that is, a period in which a conceptual image of the message is created. Critically, this initial stage in which neither the agent nor the patient were preferentially focused on is shorter than previously reported in Griffin and Bock (2000). Our participants were guided by a question referring to one of the elements, which probably led them to search for that element and construct the conceptual image promptly. Moreover, the provision of the verb might have helped them encode the action that was taking place. Note that during this period linguistic encoding has not yet started. At this point, according to our results, animacy effects are not salient, and are only marginally visible in Japanese, although they are guided by the prototypicality of the scene, as gazes were directed to a greater extent to the inanimate agent, despite it not being the HN, probably because it is a rather unlikely agent^14^. After this initial stage, speakers start the grammatical encoding of the utterance they are requested to produce. We aimed to explore whether this grammatical encoding is based on the retrieval of lexical items (*non-relational elements*) or guided by a prior structural scaffold (*relational elements*) (Konopka and Meyer, 2014). In this regard, linear incrementality accounts and hierarchical incrementality accounts make different predictions concerning the way speakers of Spanish and Japanese undertake this step and which information is prioritized in it.

If non-relational information is prioritized, we should find differences between both groups of speakers, due to opposite word order. In contrast, if relational information takes the lead, speech planning following early apprehension should be the same in both languages, since the structure to be prepared is the same.

Results showed that, despite cross-linguistic differences between Spanish and Japanese, the gaze patterns of both groups shared an identical form from 400 to 1000 ms. During this time window, gazes were directed to the HN, either the agent or the patient, depending on the type of RC Head noun. This pattern of fixation in Japanese seems to reflect the prevalence of structural over linear planning. In this language, the gaze pattern is reversed with respect to the order in which elements are going to be produced. After 1000 ms., gazes shift from the HN to the first uttered element, and then back again to the HN after 2500 ms., which is indicative of a relatively brief period of time devoted to retrieving the lexical item that will be placed in the first position. The structural representation that participants assemble in these time-windows guides subsequent gazes to both elements in the scene, so as to retrieve the corresponding lexical items in the appropriate word order. Thus, in Japanese, due to its head final nature, preparation of the structure precedes the retrieval of the lexical items in order, showing a wider scope in RC planning (see Wagner et al., 2010; Lee et al., 2013; Van de Velde and Meyer, 2014, for evidence of scope planning in complex structures). On the other hand, in Spanish, participants show the same initial pattern as Japanese speakers, with speakers focusing first on the HN. However, in Spanish the HN happens to be also the first element. With this data alone it is not possible to tell whether Spanish participants are creating a structural representation of the sentences before retrieving the corresponding lexical items, as the results suggest it is taking place in Japanese. A study by Lee et al. (2013) showed that RCs are planned as a whole in advance also in head-initial languages. In their study, they controlled the codability of the elements involved in the subordinate clause to measure the ease of speech onset. We did not control for word frequency, as it was not the aim of our study. However, we presented all the nouns and verbs to the participants beforehand, and the verb was shown again right before each picture. This likely resulted in easier access to lexical items and to the structure (see Konopka, 2012; Ganushchak et al., 2014 and Van de Velde et al., 2014, for the effects of previously seen nouns and verbs on speech planning). Nevertheless, this does not mean that structural planning was necessarily simplified or demoted in Spanish, as compared to Japanese. In any case, it seems likely that lexical retrieval started earlier in Spanish than in Japanese, in a more interwoven fashion, due to the simple fact that the Spanish grammar allows it. Thus, when there is no restriction or hindrance, speakers can make use of all the available information to be able to plan their utterance efficiently. This difference reflects the considerable degree of flexibility in language planning mechanisms (e.g., Norcliffe and Konopka, 2015), with speakers efficiently focusing their gazes on the elements they have to prepare in accordance with the structural requirements of each language [“seeing for saying,” as Bock et al. (2003) note].

Surprisingly, the role of animacy has proved to be considerably restricted in online planning. To be sure, we found clear effects in both languages in the choice of sentence types for production, with more passive than active structures when animate patients were in HN position, which confirms the finding of previous studies that animate HNs tend to be produced as subjects (of passive sentences) in RC production (Montag and MacDonald, 2009; Gennari et al., 2012). However, this is scarcely reflected in gaze patterns, both before and during linguistic encoding. Thus, it seems that the limited role of animacy in online planning is indicative of a more global planning strategy based on the overall structure of the sentence, rather than specific features of single elements. Interestingly, these results suggest as well a greater reliance on structural over thematic information, as the first focused element was regularly the HN, regardless of its being the agent or the patient of the scene.

One point of concern about the evidence of preferential looks to the HN found in both languages is that it may reflect a bias introduced by the question provided to our participants, which asked about a particular feature of the item denoted by the HN. It might thus be argued that participants were just tracing the element they were being asked about. However, based on previous findings, this possibility seems unlikely. It has been shown that speakers can locate the first information relevant to prepare the utterance in a period as short as 300 ms for a simple setting (Bock et al., 2003), or the agent and patient of a more complex action in a period as short as 400 ms. (Griffin and Bock, 2000). Hence it looks improbable that our participants took almost 1 s just to locate the item they were being asked about before engaging in linguistic encoding processes^15^. Still, we acknowledge that it is indeed difficult to ascertain to what extent linguistic encoding is involved in the period from 400 to 1,000 ms. with this data alone. Future studies should try to control for linguistic variables that might affect this time window, on the assumption that linguistic encoding is happening at this stage. Eliciting RCs without the prompt of a question, although challenging, could help clarify to what extent our participants were actually involved in planning or were rather focused on the HN in order to locate its referent.

Additionally, the possibility that initial gazes to the HN were the result of a conscious strategy in Japanese speakers, who take longer than their Spanish counterparts in starting to speak, also seems unlikely. In Study 2, with Japanese speakers, we presented an additional analysis including only speakers whose speech onset was shorter than 3 s. These “fast” Japanese speakers showed exactly the same pattern as that found when including both “slow” and “fast” participants. If any, the most remarkable difference was that “fast” speakers started speaking as soon as they prepared the first element they were going to utter (i.e., the subordinate NP), showing a more incremental planning process. These speakers are more similar to Spanish speakers in that respect.

In conclusion, the evidence reported suggests that structural information is prioritized when undertaking linguistic encoding in Japanese RC planning from the message's conceptual representation. Although further research is undoubtedly needed, these results point to a rather flexible system that allows the creation of the structure of the whole clause before retrieving the corresponding lexical items (i.e., a wider planning scope), but also allows preparation of the lexical items that are going to be produced at the same time (or at least, overlapping in time to a certain extent) as the creation of the structure in hand is taking place. It remains open to further studies the extent to, and the precise way in which structural, thematic and lexical information are related along the whole planning process, and after lexical access has started.

## Conclusions

In this study, we conducted two experiments with two typologically distant languages, in order to explore the production pattern of complex structures (relative clauses). There are, in our view, three main contributions of this study to the literature on sentence production: First, the use of relative clauses as planning targets, in contrast to simple clauses. Second, the analysis of online planning of sentences with different animacy combinations, so as to gather evidence of the role the animacy of each element plays during apprehension and linguistic planning, and how it reflects on utterance form. Finally, the comparison of Spanish and Japanese; by studying the planning process of complex structures in two typologically distant languages we aimed to clarify (1) the extent to which gazes to the first element after apprehension, widely acknowledged in the literature, represent the use of relational or non-relational information; and (2) the manner in which particular grammatical features of different languages modulate this process.

As regards the first issue, the results of the current study support a production model in which the preparation of the grammatical structure of the sentence takes the lead in comparison with the selection and retrieval of lexical items. The creation of a conceptual representation of the message allows the assembly of a structural scaffold (Bock and Ferreira, 2014), which will guide the selection of lexical elements in order. Speakers create a tentative scaffold that will allow lexical retrieval in linear order. This process does not mean that the syntactic structure is completely set and fixed before lexical retrieval starts, but allows for flexibility even after lexical retrieval has started (Rodrigo et al., 2015; see Bock and Ferreira, 2014, for a review). The flexibility of the system can be also observed between different languages, as languages in which the first element is also the most dominant one allow for the interweaving of both processes, thus making structural scaffolding and lexical retrieval more difficult to disentangle.

These results are in line with a wide array of studies that have explored the role of relational and non-relational information in different languages, in which relational information is generally prioritized, but non-relation information can be quickly used when possible (as is the case in structures in which linear and structural mappings match) (Konopka, 2012; Ganushchak et al., 2014; Van de Velde et al., 2014). However, this study provides additional evidence of the prioritizing of relational information, since, to our knowledge, it is the first one that uses a structure that allows to disentangle the effects of relational and non-relational information in the linguistic planning following the apprehension process.

In light of the conclusions gleaned from the current study, we are persuaded that the investigation of different kinds of complex structures from a cross-linguistic perspective and with an online methodology will surely continue to provide valuable evidence to assess the contribution of structural and lexical information and the interplay between them during sentence formulation.

## Ethics statement

This study was carried out in accordance with the recommendations of the Research Ethics Committee at the Universidad Autónoma de Madrid and the Human Research Ethics Committee at Hiroshima University with written informed consent from all subjects. All subjects gave written informed consent in accordance with the Declaration of Helsinki. The protocol was approved by the Research Ethics Committee at the Universidad Autónoma de Madrid and the Human Research Ethics Committee at Hiroshima University.

## Author contributions

LR contributed substantially to the design, acquisition of data, analysis and interpretation and drafting the work to be published. JI and HS contributed to the design and conception of the work, interpretation of the data and critical revisions for intellectual content. All three authors approved the final version to be published and agree to be accountable for all aspects of the work.

### Conflict of interest statement

The authors declare that the research was conducted in the absence of any commercial or financial relationships that could be construed as a potential conflict of interest.

## Footnotes

^1^Throughout this manuscript, we use the term “structural scaffold” employed by Bock and Ferreira (2014) instead of “syntactic structure” because we want to convey the notion of a syntactic plan not yet fully developed.

^2^However, see Hwang and Kaiser (2014) for an attempt to explore the timing of action planning with the creation of a region for the verb. We consider, however, that it is oftentimes difficult to determine which part of the scene corresponds to the planning of the action and isolate it from agent or patient.

^3^In addition, Japanese RCs lack relative markers (i.e., pronouns), and hence are signaled by means of case-marking particles attached to the nouns and by constituent order, namely the fact that in RCs the HN is always located after the embedded verb, as shown in Figure 2.

^4^A reviewer reminds us that in Japanese Subject RCs, the presence of a gap coindexed with the HN (see left panel of Figure 2) would result in the HN occupying an earlier position in the sentence than the subordinate-NP (the sentence-initial position, in fact). Thus, if one believes that traces are actually processing units in grammatical encoding, the comparison between Subject- and Object-RCs in Japanese would be an interesting test case for the primacy of linear order (non-relational processes) vs structural prominence (relational processes) in sentence planning.

^5^The full list of items and provided questions and verbs is available upon request to the authors.

^6^The number of responses excluded for this reason was only one out of 1,468 (0.07 % of valid responses).

^7^Variations of the same type of constructions were accepted (e.g., responses with different lexical items that conveyed the same meaning).

^8^The 31.91% of responses excluded for gaze pattern analysis were all grammatical and well-formed sentences in Spanish: they consisted mainly of passive sentences with the by-phrase dropped or sentences that did not begin with the HN (e.g., *Is wearing a pink blouse the girl who pushes the boy*, instead of *The girl who pushes the boy is wearing a pink blouse*). These sentences, however natural and correct in Spanish, would have made it difficult to control the timing of gaze patterns and draw conclusions for them. Thus, despite the high amount of excluded responses resulting from these stringent criteria, we deemed it was the best solution to improve gaze pattern analysis. The same applies for experiment 2.

^9^Spanish allows sentences in which the subject is not overtly expressed, while the object remains in place. In these sentences, the agent is implicitly referred to by a plural inflection in the verb (e.g. “El soldado al que están empujando” – The soldier whom (they) are pushing).

^10^Note that the mean naming latency varies between Spanish and Japanese. In Spanish, mean RT to Snodgrass and Vanderwart picture set is 829 ms. (Cuetos et al., 1999), while in the case of Japanese mean RT is 1148 ms. (Nishimoto et al., 2005). In order to ensure direct comparison between Spanish and Japanese, we chose a middle point for setting the second time window.

^11^Based on a suggestion made by one of the reviewers, in this figure we decided to collapse data from Spanish (Experiment 1) and Japanese (Experiment 2) on the overall pattern of gazes to the agent and to the patient, in order to facilitate comparison across languages.

^12^In Japanese, contrary to Spanish, starting from the main clause (i.e. “is wearing a read T-shirt”), instead of the RC under analysis, is much less common, when the RC is modifying the subject of the main clause, as is the case in this study. Thus, there were fewer sentences excluded for gaze analysis in Japanese.

^13^This pattern of results occurs regardless of the position of potential gaps in Subject- and Object-RCs (see footnote 4 above).

^14^See Rosen (1999) for an analysis of the constraints on animacy and agency in Japanese in contrast to Spanish or English. These constrains could have an influence in making inanimate doers a more unlikely agent for Japanese speakers than for Spanish speakers in our task.

^15^Ganushchak et al. (2014) also conducted an experiment in which they focused their participants' attention by providing a question: e.g., “What is the policeman stopping?” Although their results show some differences with respect to ours, they also indicate that the search of the object that was being asked about took place quickly and efficiently. One of the main differences between their results in Dutch (object-focus, which is the most similar condition to our RCs in Japanese) and our results is that in their case, participants barely looked at the subject once they created the structural scaffold. This is so because the lexical item for the subject (“the policeman”) was provided in the question, so that name-related gazes were not needed. Note that we did not include the name label of any of the elements in the questions.
